# Peace, equanimity and acceptance in the cancer experience: validation of the German version (PEACE-G) and associations with mental health, health-related quality of life and psychological constructs

**DOI:** 10.1186/s40359-024-02018-8

**Published:** 2024-09-27

**Authors:** Christina Sauer, Till Hansen, Holly G. Prigerson, Jennifer W. Mack, Till J. Bugaj, Gregor Weißflog

**Affiliations:** 1 Department of General Internal Medicine and Psychosomatics, University Hospital Heidelberg, Heidelberg, Germany; 2 National Center for Tumor Diseases (NCT), University Hospital Heidelberg, Heidelberg, Germany; 3 Institute of Medical Psychology, University Hospital Heidelberg, Heidelberg, Germany; 4 Cornell Center for Research on End-of-Life Care, Weill Cornell Medicine, New York City, NY USA; 5 Department of Pediatric Oncology, Division of Population Sciences, Dana-Farber Cancer Institute, Boston, MA USA; 6 Department of Medical Psychology and Medical Sociology, University of Leipzig, Leipzig, Germany; 7 Comprehensive Cancer Center Central Germany (CCCG) Leipzig-Jena, University Medical Center Leipzig, Leipzig, Germany

**Keywords:** Psycho-oncology, Cancer, Acceptance, Validation, Coping, Emotion regulation, Mindfulness

## Abstract

**Background:**

Systematic reviews and meta-analyses reveal the importance of an accepting attitude towards cancer for mental health and functional coping. The aim of this study was to examine the psychometric properties of the German translation of the Peace, Equanimity, and Acceptance in the Cancer Experience (PEACE) questionnaire (Mack et al., 2008) and to investigate its associations with mental health, health-related quality of life (HRQoL) and related constructs.

**Methods:**

The German version of the PEACE (PEACE-G) was created and validated with cancer patients in a cross-sectional two center questionnaire study. Construct validity was tested with confirmational factor analyses (CFA); Cronbach’s alpha was used to determine internal consistency of items. We further examined associations with depression (PHQ-8), anxiety (GAD-7), distress (NCCN distress thermometer), HRQoL (SF-12), psychological flexibility (AAQ-II), resilience (RS-11) and acceptance scales, to evaluate concurrent and divergent validity.

**Results:**

*N* = 213 cancer patients with different tumor entities participated in this study. Results of the CFA replicated the two-factor solution of the original PEACE (peaceful acceptance and struggle with illness) with satisfactory psychometric properties. Peaceful acceptance showed negative associations with depression, anxiety, distress, psychological inflexibility, and positive associations with HRQoL, acceptance, resilience, and mindfulness. Results of sensitivity analyses indicate a third factor (injustice/anger).

**Conclusions:**

The results demonstrate the validity and reliability of PEACE-G in cancer patients and point to the essential role of acceptance- and mindfulness-based interventions in psycho-oncology. Further studies are needed to investigate the different facets of struggle with the cancer illness.

**Supplementary Information:**

The online version contains supplementary material available at 10.1186/s40359-024-02018-8.

## Background

Cancer patients face a variety of biopsychosocial stressors. They must cope with the life-threatening diagnosis, side effects of the therapy, diverse losses, and reduced functionality. Functional coping with these distressing factors is pivotal to improve mental health and health-related quality of life (HRQoL) of cancer patients [e.g., 1]. Psycho-oncological interventions are empirically supported methods for alleviating psychological distress and enhancing both mental health and HRQoL [e.g., 2, 3, 4].

A coping strategy deemed crucial in the effective management of cancer and its consequences is the acceptance of cancer and its sequelae. Accepting negative thoughts and emotions can happen by integrating the illness into one’s identity, embracing uncertainty, being aware of the present moment, and aligning one’s life with values and what is important despite cancer. Several conceptualizations of cancer acceptance exist, that are included in the coping theory of Lazarus and Folkman [5], meaning making model [6] or theories of third wave cognitive behavioral therapies, e.g., Acceptance and Commitment Therapy (ACT) [7]. Recently, Secinti et al. [8] developed an integrated model of cancer acceptance, combining the ideas of the aforementioned theories. They defined cancer acceptance as the ‘active willingness to be present with cancer-related realities while giving up efforts to judge or control cancer-related appraisals or feelings. In addition, cancer acceptance involves a behavioral willingness in response to cancer-related stressors, resulting in actions aligned with deeply held values.’ [8, p. 29].

It is important to distinguish cancer acceptance from resignation or fatalism [8]. Resignation entails regarding the illness as an inevitable fate, wherein one believes there is minimal or no capacity to alter or manage the illness, its symptoms, and one’s overall HRQoL. Additionally, acceptance differs from mere tolerance or adopting a fighting spirit, which involves perceiving the illness as a challenge, maintaining an optimistic perspective, and actively striving to overcome the disease.

The opposite of acceptance is experiential avoidance, which refers to attempts to alter or control the intensity, frequency or form of internal experiences [9]. A recent systematic review in patients with advanced cancer revealed that experiential avoidance is associated with several negative outcomes, e.g., reduced HRQoL, increased distress, and disturbed mood [10]. This pattern is also shown in cancer patients with curable disease [11, 12]. Experiential avoidance is also an important predictor, mediator, and moderator for emotional distress and HRQoL in cancer patients [13–15].

Meta-analytic reviews underline the importance of cancer acceptance. It was inversely associated with general and cancer-related distress, depression, and anxiety, with small to moderate effect sizes [8]. Further analyses showed that the association between acceptance and depressive symptoms was stronger in older patients, and that the association of acceptance and depressive symptoms as well as anxiety symptoms was stronger in early-stage patients and patients without a partner or spouse. In a recent meta-analysis, acceptance was negatively correlated with distress, with a small effect size (*r* = -.24) [16]. However, acceptance was assessed with the COPE inventory, which assesses coping with the cancer diagnosis itself rather than the specific process of living with cancer. Research on cancer acceptance and its impact on patients’ mental health and HRQoL requires specific, valid, and reliable measures to capture the extent to which patients have accepted and come to terms with the disease (i.e., peaceful acceptance) or are struggling to do so (in the sense of experiential avoidance).

The Peace, Equanimity, and Acceptance in the Cancer Experience (PEACE) questionnaire measures to which extent cancer patients can accept their illness or if they struggle with it [17]. The questionnaire consists of 12 items and comprises two subscales. The *peaceful acceptance* (PA) subscale (five items) describes to which extent patients can accept and make peace with the cancer diagnosis, have a sense of inner peace, harmony, and equanimity, and believe that they are loved. The *struggle with illness* (SwI) subscale (seven items) measures to which extent patients have difficulties living from day to day because of worrying about the illness, to which extent they are upset by changes in their physical appearance, and if they feel like the illness has beaten them down. Both subscales are negatively correlated. The PEACE showed good reliability of the total scale (Cronbach’s α = 0.85) and of both subscales (Cronbach’s α (*PA*) = 0.78; Cronbach’s α (*SwI*) = 0.81). The sample of the original study comprised patients with advanced cancer (expected prognosis < one year) [17].

So far, the PEACE was translated into Japanese and validated in cancer patients (all stages and various entities) [18]. This study provided the first evidence of construct validity via exploratory factor analyses (EFA) and confirmatory factor analyses (CFA) for the PEACE. Results confirmed the factor structure of the original PEACE with an adequate fit [18]. The reliability for the subscales was very good with *α* = 0.84 for PA and α = 0.86 for SwI. The intercorrelation between both subscales was *r* = -.49 (*p* < .01) in Japanese cancer patients of all stages, a level comparable to *r* = -.54 (*p* < .001) found in the original PEACE study with advanced cancer patients [17].

The aims of this study were to (1) translate the PEACE into German, (2) to examine the construct and convergent validity and the reliability of the German version of the PEACE (PEACE-G), and (3) to investigate the association between the subscales of the PEACE-G and psychological burden, HRQoL, and related psychological constructs (i.e., psychological flexibility, mindfulness, resilience). Because of the need for research on the validity of acceptance as a construct, this study examines the factor structure of the PEACE-G and thus sheds light on what latent factors are involved in cancer acceptance.

## Methods

In this two-center cross-sectional study we recruited cancer patients at the National Center for Tumor Disease (NCT), the Cancer Counselling Service Heidelberg, and Leipzig University Hospital during November 2022 and February 2023. Research assistants informed patients about the study and asked for participation. Patients filled out the questionnaires after informed consent was obtained. The study was conducted either online via SosciSurvey on tablets, after scanning a QR code on patients’ smartphone, or with a paper-pencil version. The study was approved by the Ethics Committees of the Medical Faculties in Heidelberg (S-494/2022) and in Leipzig (336/22-lk) and was conducted in accordance with the Declaration of the Helsinki.

### Participants

Inclusion criteria were a cancer diagnosis of any entity and stage, age ≥ 18 years, German as the mother tongue or excellent knowledge of German, and written declaration of consent from. Exclusion criteria included the presence of a cognitive or physical impairment that makes it difficult to complete the questionnaires (e.g., impaired eyesight) or severe psychiatric illness (e.g., psychosis or dementia). Before engaging with patients at the NCT Heidelberg day clinic, the internal clinical system was screened to identify any arrivals scheduled for treatment that day who met the exclusion criteria. At the other recruitment sites, a clinical psychologist checked the inclusion and exclusion criteria.

### Translation of the PEACE

The PEACE was translated into German language following the *Translation*,* Review*,* Adjudication*,* Pretest*,* Documentation* (TRAPD) method of the GESIS Survey Guidelines [19]. It was first translated independently by two researchers (CS and GW) with very good command of English, and one person with a professional command of English. The three versions were discussed, open questions were clarified with an adjudicator, and a consensus version was constructed. In addition, the original authors (HP and JM) were contacted to clarify the meaning of special words and to slightly change the structure of the questions. After this step, the version was retranslated by a native speaker. This version was then compared with the original PEACE and the final version was created (see Supplement 1).

### Measures

We assessed the following questionnaires and additional sociodemographic and medical information. Where medical information was missing, data was added from medical records in the internal clinical documentation system. Each patient received a pseudonymized code that did not contain their initials or date of birth.

Patient-Health-Questionnaire-8 (PHQ-8): Depressive symptoms were assessed using the PHQ-8 (i.e., the PHQ-9 without the suicidal ideation item) [20]. The PHQ-8 shows equivalent diagnostic accuracy as the PHQ-9 [21, 22]. Higher values indicate more severe symptoms. Cronbach’s *α* in our study was = 0.81.

Generalized Anxiety Disorder Questionnaire (GAD-7): Symptoms of generalized anxiety were measured with the GAD-7 [23]. The German version shows good factorial and construct validity as well as good internal consistency [23]. Cronbach’s *α* in our study was = 0.88.

National Comprehensive Cancer Network (NCCN) distress thermometer: The German version of the NCCN distress thermometer [24] was used to assess psychosocial distress.

Short-Form-Health-Survey-12 (SF-12): We assessed HRQoL with the SF-12, the short version of the SF-36 Health Survey [25]. The SF-12 provides two scales: a physical scale score and a psychological scale score. The German version of the SF-12 shows high reliability and validity [26]. Internal consistency was not calculated for SF-12, because of the different levels of measurement of items.

Acceptance and Action Questionnaire-II (AAQ-II): We measured psychological inflexibility with the German version of the AAQ-II [27, 28]. The AAQ-II consists of seven items and showed good validity and reliability [27, 28]. Cronbach’s *α* in our study was = 0.91.

Five Facet Mindfulness Questionnaire (FFMQ-D): We used the *Accept without Judgement* and the *Act with Awareness* scale of the German version of the FFMQ-D, both subscales showing good internal consistency [29]. In our study, Cronbach’s *α* was = 0.92 for the *Accept without Judgement* scale, and = 0.87. for *Act with Awareness* scale.

German Resilience Scale (RS-11): The RS-11 is a short scale of the original RS-25 with 11 items and has proven to be a reliable measure for the assessment of resilience as a trait [30]. Cronbach’s *α* in our study was = 0.93.

Acceptance Scale of the Emotion Regulation Skills Questionnaire (SEK-27): We used the three-item acceptance scale of SEK-27, which also showed satisfactory internal consistency as subscale [31]. Cronbach’s *α* in our sample was = 0.81.

### Analysis

Descriptive statistics and correlation analysis were conducted with SPSS 27. Missing data were excluded pairwise. For all analyses, we considered a significance level of *p* < .05 as statistically significant. Outlier analyses showed one case with a *z*-standardized value of *z* > |3.29| in several scales. The analyses were calculated with and without this case. Since the results did not differ, it was subsequently included in all analyses. Differences in the PEACE-G subscales related to different sociodemographic variables were tested using Spearman rank correlation for ordinal scaled variables, nonpaired t-tests (independent samples) for binary nominal scaled or ANOVA for nominal scaled variables with more than two groups, and Pearson correlation for continuous variables. To determine convergent validity, correlations between the two PEACE-G subscales and the validation questionnaires were calculated. Spearman rank correlation was used for those with a deviating distribution. Each scale deviated significantly from normal distribution (*p* < .01) according to the Shapiro-Wilk test. Given the large sample size’s influence on significance testing, this was further assessed through histogram analysis to justify assuming approximate normality, enabling Pearson correlations calculation. In the case of the two FFMQ scales and the FAH-2 scale, no normal distribution was graphically apparent, so the Spearman rank correlation was calculated. To assess internal consistency, reliability was calculated using Cronbach’s α and item-scale correlation was examined. Cronbach’s α was interpreted according to common cutoffs for a good α, which range from 0.70 to 0.80 [32, 33].

CFA using SPSS Amos 27 tested the original PEACE factor structure, with PA and SwI forming distinct yet correlated dimensions of acceptance. The structural equation model (SEM) featured two intercorrelated latent factors explaining item variance: one influencing items 1–5 (PA) and the other, items 6–12 (SwI). Residual variances were adjusted for each item, with modification indices guiding model refinement. A covariance between residuals of items 8 and 10 was incorporated for improved fit. Single-sample cross-validation assessed model stability by partitioning the sample into two subsamples and comparing model improvements resulting from this covariance integration. The variance of all latent variables was standardized and fixed at 1, which is recommended when investigating the dimensionality of the items [34]. Following common conventions for evaluating model fit indices, a CFI value ≥ 0.90 indicates an acceptable model fit [35]. A Root Mean Square Error of Approximation (RMSEA) value ≤ 0.05 can be described as a good fit, 0.05 < RMSEA < 0.08 as an adequate fit, and 0.08 < RMSEA < 0.10 as a mediocre fit, whereas values ≥ 0.10 are beyond acceptable [36].

Several sensitivity analyses were conducted to investigate possible alternative model structures to improve the model fit (EFA with principal axis analysis (PAA), SEM with one-, bi-factor- and three-factor-model; see Supplement 2 and 3 for a detailed description).

## Results

### Sample

*N* = 230 cancer patients consented to participate in this study. *N* = 17 patients had to be excluded due to not filling out any of the questionnaires after registration (*n* = 12) or not filling out most of the questionnaires (*n* = 1), not fulfilling the inclusion criteria of a cancer diagnosis (*n* = 2), due to more than one answer in the PEACE-G items (*n* = 1), and one participant stated after the survey that he/she could not answer the questions honestly.

Final sample size comprised *N* = 213 participants, which were recruited in Heidelberg (97.4%) and Leipzig (2.6%)^1^. 86% of the participants completed the survey online via tablet or smartphone, 14% chose to fill out the paper-pencil version. 64% of the sample were female, and the mean age was 54.24 years (*SD* = 11.50, range = 19–80). The mean time since diagnosis was 3.09 years (*SD* = 4.10 years).

Further sociodemographic characteristics of the sample are presented in Table 1, medical characteristics are presented in Table 2.

**Table 1:** Sociodemographic sample characteristics

Patient characteristics	*n* (%)
German nationality^a^	202 (94.8)
Population size of residential area	
100.000	38 (17.8)
20.000–100.000	36 (16.9)
5.000–20.000	69 (32.4)
< 5.000	70 (32.9)
Marital status^a^	
Single	40 (18.9)
Married, living together	139 (65.6)
Married, not living together	8 (3.8)
Divorced	21 (9.9)
Widowed	4 (1.9)
Participants living in a partnership	178 (83.6)
Highest school grade	
Main school certificate	45 (21.1)
Secondary school certificate	77 (36.2)
Abitur^c^	83 (39.0)
Other / None	8 (3.8)
Employment	
Employee	113 (53.1)
Self-employed	20 (9.4)
Civil servant	13 (6.1)
Unemployed	5 (2.3)
In training	1 (0.5)
Retired	46 (21.6)
Other	15 (7.0)
Sick leave^b^	92 (43.2)
Income (*n* = 207; € monthly)	
< 250	1 (0.5)
250–500	5 (2.3)
500–1000	21 (9.9)
1000–1500	36 (16.9)
1500–2000	38 (17.8)
2000–3000	46 (21.6)
3000–4000	36 (16.9)
4000–5000	9 (4.2)
> 5000	11 (5.2)
no comment	4 (1.9)

Note. *N* = 213.^a^*n* = 212 ^b^*n* = 211. ^c^Abitur is the highest school leaving qualification in Germany

**Table 2:** Participants’ medical characteristics

Characteristic	*n* (%)
Type of cancer	
Breast	85 (39.9)
Malignant melanoma	32 (15.0)
Colorectal	17 (8.0)
Pancreatic	14 (6.6)
Stomach	8 (3.8)
Kidney	8 (3.8)
Oesophageal	7 (3.3)
Head and neck	5 (2.3)
Liver	5 (2.3)
Cervical	4 (1.9)
Ovarian	4 (1.9)
Blood and lymph gland	3 (1.4)
Neuroendocrine	3 (1.4)
Penis or testicular	3 (1.4)
Cholangiocarcinoma	2 (0.9)
Prostate	2 (0.9)
Lung	2 (0.9)
Bladder	1 (0.5)
Other	8 (3.8)
First cancer diagnosis	164 (77.0)
Current treatment (multiple choice)	
Chemotherapy	148 (69.5)
Immunotherapy	83 (39.0)
OP	9 (4.2)
Radiotherapy	8 (3.8)
Metastatic cancer	126 (59.2)

*Note: N* = 213

### Confirmatory factor analysis

**Fig. 1:**
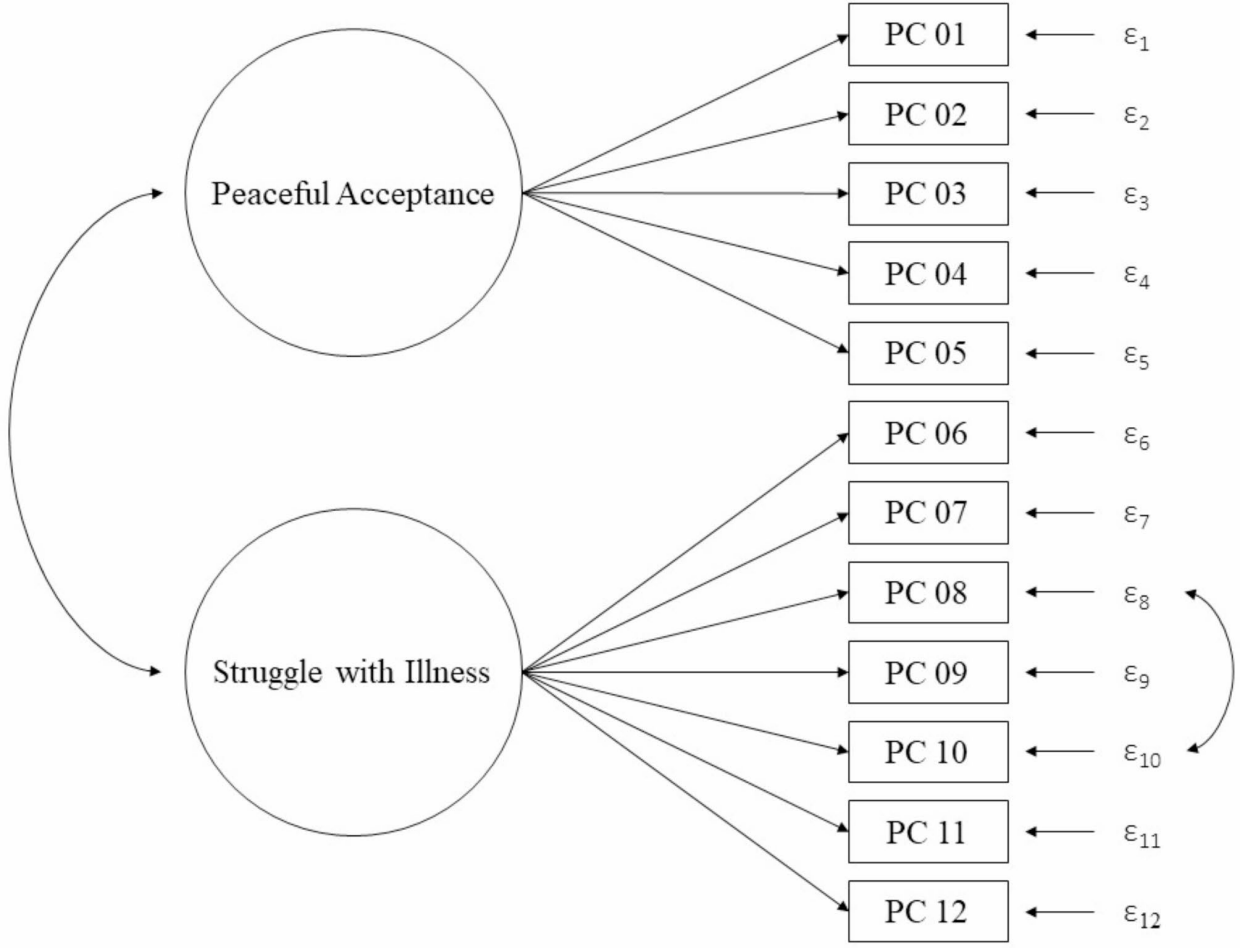
Final SEM of the PEACE-G

Incorporating a covariance between the residuals of PC 08 and PC 10 resulted in the final model, which showed satisfactory outcomes (χ² (df) = 123.70 (52), *p* < .001, CFI = 0.918, RMSEA (90% CI) = 0.081 (0.062–0.099)), with moderate RMSEA and acceptable CFI values. An illustration of the final model is shown in Fig. 1.

Results of the single-sample cross-validation indicated that the integration of the covariance between the residuals of PC 08 and PC 10 led to a substantial model fit improvement in each subsample constellation (see Supplement 4).

### Convergent validity

The correlation between the *PA* and *SwI* subscale was *r* = − .64, *p* < .01. Correlation coefficients of *PA* and *SwI* with symptoms of psychological distress and related constructs are depicted in Table 3. All correlation coefficients reached significance. PA showed negative associations with depression, anxiety, distress, psychological inflexibility, and positive associations with HRQoL, acceptance, resilience, and mindfulness, with small to large effect sizes. *SwI* showed inverse significant correlations with the constructs examined.

**Table 3:** Correlation between the PEACE subscales and psychological burden, HRQoL and related constructs

	FFMQ - AOB^a^	FFMQ - MAH^a^	RS-11	SEK-A	SF-12 - physical	SF-12 - psych	PHQ-8	GAD-7	Distress	AAQ-II^a^
*Peaceful Acceptance*	0.38***	0.40***	0.37***	0.40***	0.14*	0.56***	− 0.53***	− 0.56***	− 0.44***	− 0.57***
*Struggle with Illness*	− 0.33**	− 0.40**	− 0.33***	− 0.20**	− 0.30***	− 0.50***	0.56***	0.53***	0.45***	0.54***

*Note.* ***p < .001. **p < .01. *p < .05. ^a^Spearman correlation coefficient

Note: FFMQ-AOB = Five Facet Mindfulness Questionnaire *Accept without Judgement* scale. FFMQ - MAH = Five Facet Mindfulness Questionnaire *Act with Awareness* scale. RS-11 = German Resilience Scale; SEK-A = Acceptance scale of the *Fragebogen zur Standardisierten Selbsteinschätzung Emotionaler Kompetenzen*. SF-12 = Short-Form-Health-Survey-12, physical and psychological scale score. PHQ-8 = Patient-Health-Questionnaire-8; GAD-7 = Generalized Anxiety Disorder Questionnaire. Distress = assessed with NCCN distress thermometer; AAQ-II = Acceptance and Action Questionnaire-II

### Reliability

Cronbach’s α of the total scale was α = 0.86, the subscales showed a Cronbach’s α of 0.79, each, indicating good reliability.

As an additional analysis of item parameters, the item-scale correlations were calculated. Except for item 4 (‘Do you feel loved now?’, *r* = .26), the correlations were all between *r* = .43-0.70. The correlations and the level of Cronbach’s α when an item is excluded from the scale are depicted in Supplement 5.

### Sensitivity analysis

The *PA* and *SwI* scores had no relation with sex, nationality, marital status, children, children living at home, number of inhabitants of hometown, level of school and professional education, employment status, income level, and all medical information. Patients on sick leave reported significantly lower *PA* (*M* = 15.22, *SD* = 2.90) than patients not on sick leave (*M* = 16.53, *SD* = 2.82; *t*(207) = -3.30, *p* < .01, *d* = -0.46), and higher *SwI* (*M* = 17.57, *SD* = 4.39) than patients not on sick leave (*M* = 15.42, *SD* = 4.65; *t*(209) = 3.42, *p* < .01, *d* = 0.48). *PA* correlated significantly with age; *r* = .18, *p* < .05.

Results of EFA indicated three factors (see Supplement 2). In this model, the items of the *SwI* scale are distributed to two factors, with PC 08 and PC 10 loading remarkably high on factor 2; PC 07, PC 09, PC 11, and PC 12 loading on factor 3; and PC 06 loading almost comparably on factor 2 and 3 (see Table S2-1, Supplement 2 for a detailed description). Based on these results we conducted a CFA with three factors. The model comprised a *PA* factor (PC 01–05), a second factor (PC 08, PC 10), and a third factor (PC 06, PC 07, PC 09, PC 11, PC 12). The three-factor-model yielded better fit indices than the two-factor solution (see Figure S3-1, Supplement 3). However, the slight improvement in indices comes at the cost of reduced parsimony and simplicity. Further, at least three items should load on a factor [37]. Thus, we decided to remain at the described two-factor solution.

Further sensitivity analyses indicated a substantial better model fit in the subsample of patients with non-metastatic cancer versus patients with metastatic cancer. SEMs of alternative models did not improve the model fit. Results of these models are depicted in Supplement 3.

## Discussion

This study investigated PA and SwI and its associations with mental health, HRQoL and psychological constructs like resilience, psychological flexibility, and mindfulness. We found significant associations of both scales with all investigated constructs. Further, results show that the PEACE-G is a reliable and valid measure to assess acceptance in the cancer experience. Our findings underline the importance of acceptance in the context of cancer as well as of psycho-oncological acceptance- and mindfulness-based interventions.

The significant correlations of both PEACE-G scales with HRQoL, psychological distress and related constructs underscore the positive effect of an accepting attitude in the context of cancer. In previous meta-analytic reviews, depressive symptoms, anxiety, and psychological distress showed correlations with acceptance with small to medium effect sizes (*r* = − .22 to *r* = − .31) [8] or small effect size (*r* = − .24) [16]. In our study, associations showed medium to small effects (*r* = − .44 to − 0.56). One reason for this may be methodological, as the reviews combined different measures of acceptance that may capture different aspects of acceptance, whereas this study assessed acceptance within the cancer experience, which is even more specific for cancer patients.

Regarding the validation of the PEACE-G, satisfactory construct validity was shown by confirming the model fit of the two-factor structure of the original PEACE [17] with an integrated covariance between item 8 (‘To what extent do you feel that it is unfair for you to get cancer now?’) and item 10 (‘To what extent do you feel angry because of your illness?’). The fit indices are comparable to the indices of the Japanese version of the PEACE [18]. There are two possible explanations for a better model fit with covarying residual variances. First, this might be explained by a sample artefact. However, cross-validation showed that the covarying residuals provided better model fits underlining the stability of the results. A second explanation is a systematic source of variance not yet integrated into the model [38]. In line with this, results of the EFA provide important information about potential facets of SwI. PAA identified three factors with Eigenvalues > 1, indicating an additional dimension influencing cancer acceptance beyond PA and SwI, with PC 08 and PC 10 loading significantly on a factor associated with perceived injustice and anger towards cancer illness. Perceived injustice, a cognitive assessment, reflects a feeling of unfairness linked with various emotional reactions. Our findings imply that perceived injustice and anger might not be related to SwI, as measured by other items like concerns about physical appearance. A recent study found negative associations between perceived injustice and cancer acceptance and positive associations with psychosocial outcomes, including depression, anxiety, and anger [39]. In their model, the association between perceived injustice and psychosocial outcomes was mediated by acceptance (not meaning making), suggesting that acceptance is the central mediating process in this adjustment process. Thus, perceived injustice as a cognitive appraisal seems to be something to be dealt with in acceptance rather than an aspect of coping with illness. Longitudinal studies are needed to investigate the causality of these processes.

The reliability of the total scale and the two subscales yields good Cronbach’s alpha, which is in line with the results of the original and the Japanese version [17, 18]. The finding that the consistency of the total scale is higher than that of the subscales may indicate that both subscales assess the same constructs (PA). However, since the one-factor model shows a worse model fit than the two-factor model, it seems that both factors are justified.

The relationship between age and acceptance of cancer stands in line with findings of previous research [8, 17]. This may be due to older patients, particularly those in late adulthood, being more accepting of mortality compared to younger patients, as the inevitability of death becomes more apparent with age. That neither sex, partnership, nor time since diagnosis affects acceptance was also shown in a meta-analytic review [8]. The association between sick leave and acceptance may be because patients with a milder course and less aggressive cancer are less likely to be sick.

This study revealed that the PEACE-G scale effectively measures cancer acceptance in patients in the curative situation, a factor not previously addressed in the original study [17]. The better model fit in the sub-sample of patients with non-metastatic cancer suggests that it tends to measure acceptance even better in this sample.

Several alternative models were calculated, none of which resulted in substantially better model fits. Following the principle of Occam’s razor, which says that entities should not be multiplied beyond necessity, the three-factor-model did not improve model fit enough to abandon the parsimony and consistency of a two-factor solution. However, the possibility of an additional source of variance from PC 08 and PC 10 is accounted for in the final model by integrating the covariance. Finally, there is evidence to assume that the two-factor-model of the original PEACE is also the best explanation for the PEACE-G.

### Clinical implications

The high correlations between PA and SwI with psychological symptoms, HRQoL, resilience, psychological flexibility, and mindfulness, underline the importance of acceptance for mental health and HRQoL. These results are in line with previous studies showing the importance of acceptance of cancer for mental health in patients with cancer [39]. Therefore, psycho-oncologists should explore the acceptance of cancer and emotions in psychologically burdened cancer patients.

Further, the results underpin the importance of acceptance-based interventions in psycho-oncology. For example, ACT [40], a third-wave cognitive-behavioral approach, promotes acceptance and value-based living, while increasing distance from dysfunctional thoughts and emotions. Results from an umbrella review revealed that ACT increases HRQoL and reduces depression and anxiety, but more high-quality studies are needed [41].

Further, mindfulness-based interventions might be promising to increase acceptance of cancer and emotions as well as HRQoL, and to reduce psychological burden. Mindfulness includes an acceptance stance towards all experiences per definition. Meta-analytic reviews show the positive effects of mindfulness interventions in cancer patients on various outcomes [42, 43], but there are no studies investigating the mediating role of acceptance in this context.

### Study limitations

The results of this study must be seen in the context of its limitations. We conducted a cross-sectional study that did not allow to examine retest reliability or sensitivity for change. Thus, the extent to which the PEACE-G may also capture changes over time or be a reliable measure of cancer acceptance at different time points, was not investigated in this study. Considering that acceptance of the disease is a psychological adjustment and process, this question could be of interest. In this context, it has also been suggested that the PEACE may not measure aspects of peacefulness that are responsive to interventions [44]. It is possible, for example, that the PEACE rather measures a stable trait and less a state that is susceptible to change. Future studies are needed to address this aspect. Furthermore, the sample was non-homogeneous with regard to diagnosis and cancer severity, and no conclusions can be drawn about the causality of the effects due to the cross-sectional design.

## Conclusion

The results of this study underline the importance of illness acceptance in the context of cancer. The associations between mental health, HRQoL, psychological flexibility, mindfulness, and resilience indicate that greater acceptance and reduced struggle are important factors in the illness management. Further, this study showed that the PEACE-G is a reliable and valid instrument. It can be used in research and clinical practice. Our results indicate the possibility of a three-factor solution of the construct. In this case, the construct of acceptance would be more like a triangle of three factors that can influence each other but can also act independently of each other: Acceptance - Struggle – Perceived Injustice/Anger. In sum, the results underline the importance to explore an acceptance stance and to offer acceptance-based interventions in patient with cancer to improve HRQoL and mental health.

## Electronic supplementary material

Below is the link to the electronic supplementary material.

Supplementary Material 1

Supplementary Material 2

Supplementary Material 3

Supplementary Material 4

Supplementary Material 5

## Data Availability

Data of this study are available from the corresponding author upon reasonable request.

## Footnotes

1 The unbalanced recruitment in the two study centers resulted from the fact that two master students collected the data there, and not due to sampling bias.

## Abbrevations

AAQ-II: Acceptance and Action Questionnaire-II
ACT: Acceptance and commitment therapy
CFA: confirmational factor analyses
EFA: exploratory factor analyses
FFMQ - MAH: Five Facet Mindfulness Questionnaire Act with Awareness scale.
FFMQ-AOB: Five Facet Mindfulness Questionnaire Accept without Judgement scale.
GAD-7: Generalized Anxiety Disorder Questionnaire
HRQoL: health-related quality of life
NCCN: National Comprehensive Cancer Network
NCT: National Center for Tumor Disease
PA: peaceful acceptance
PAA: principal axis analysis
PEACE: Peace, Equanimity and Acceptance in the Cancer Experience
PEACE-G: German PEACE
PHQ-8: Patient-Health-Questionnaire-8
RMSEA: Root Mean Square Error of Approximation
RS-11: German Resilience Scale;
SEM: structural equation model
SwI: struggle with illness
TRAPD: Translation, Review, Adjudication, Pretest, Documentation
