# Supplementary material for: Peace, equanimity and acceptance in the cancer experience: validation of the German version (PEACE-G) and associations with mental health, health-related quality of life and psychological constructs
Source: BMC Psychol. 2024 Sep 27;12:507. doi: 10.1186/s40359-024-02018-8 (PMC11438294; doi:10.1186/s40359-024-02018-8)
Supplement: Supplementary file 1 — Supplementary Material 1 [file 40359_2024_2018_MOESM1_ESM.docx]

**Supplement 1**

*PEACE-G*

| Kreisen Sie die Antwortmöglichkeit ein, die am besten beschreibt, wie Sie sich gerade fühlen: | überhaupt nicht | kaum | etwas | sehr |
| --- | --- | --- | --- | --- |
| 1. Können Sie Ihre Krebsdiagnose akzeptieren?   [‘To what extent are you able to accept your diagnosis of cancer?‘] | 1 | 2 | 3 | 4 |
| 1. Empfinden Sie Ihrer Meinung nach inneren Frieden und Harmonie?   [‘To what extent would you say you have a sense of inner peace and harmony?] | 1 | 2 | 3 | 4 |
| 1. Haben Sie Frieden mit Ihrer Erkrankung geschlossen?   [‘To what extent do you feel that you have made peace with your illness?’] | 1 | 2 | 3 | 4 |
| 1. Fühlen Sie sich derzeit geliebt?   [‘Do you feel well loved now?’] | 1 | 2 | 3 | 4 |
| 1. Empfinden Sie innere Ruhe und Gelassenheit?   [‘To what extent do you feel a sense of inner calm and tranquility?’] | 1 | 2 | 3 | 4 |
| 1. Belasten Sie Veränderungen Ihres äußeren Erscheinungsbildes?   [‘To what extent do changes in your physical appearance upset you?’] | 1 | 2 | 3 | 4 |
| 1. Hindert Sie die Sorge über Ihre Erkrankung, unbeschwert durch den Tag zu kommen?   [‘To what extent does worry about your illness make it difficult for you to live from day to day?’] | 1 | 2 | 3 | 4 |
| 8. Empfinden Sie es unfair, jetzt an Krebs erkrankt zu sein? [‘To what extent do you feel that it is unfair for you to get cancer now? ‘] | 1 | 2 | 3 | 4 |
| 9. Haben Sie das Gefühl, dass Ihr Leben, so wie Sie es kennen, jetzt vorbei ist? [‘To what extent do you feel that your life, as you know it, is now over? ‘] | 1 | 2 | 3 | 4 |
| 10. Sind Sie verärgert über Ihre Erkrankung? [‘To what extent do you feel angry because of your illness? ‘] | 1 | 2 | 3 | 4 |
| 11. Fühlen Sie sich von der Erkrankung in die Knie gezwungen? [‘To what extent do you think your illness has beaten you down? ‘] | 1 | 2 | 3 | 4 |
| 12. Schämen Sie sich für Ihren derzeitigen Gesundheitszustand bzw. ist Ihnen dieser peinlich? [‘To what extent do you feel ashamed of, or embarrassed by, your current condition?‘] | 1 | 2 | 3 | 4 |

***Note:*** Items of the PEACE-G with the English original PEACE items.
