# Supplementary material for: Peace, equanimity and acceptance in the cancer experience: validation of the German version (PEACE-G) and associations with mental health, health-related quality of life and psychological constructs
Source: BMC Psychol. 2024 Sep 27;12:507. doi: 10.1186/s40359-024-02018-8 (PMC11438294; doi:10.1186/s40359-024-02018-8)
Supplement: Supplementary file 2 — Supplementary Material 2 [file 40359_2024_2018_MOESM2_ESM.docx]

**Supplement 2: Results of explorative factor analysis**

Explorative Factor Analysis (EFA)

We performed EFA to explore potential alternative model structures aimed at enhancing the overall model fit. Following the guidelines of Watkins (2018), a principal axis analysis (PAA) with least-square estimation method was used and the solution was rotated via Promax. The resulting structure matrix shows correlations between factor and variable, the pattern matrix shows these correlations after partialing out influences of the other correlated factors and is recommended to be evaluated first (Watkins, 2018). The scree plot, pattern matrix, explained variances and structure matrix are displayed in figure an tables below. Results of the pattern matrix and graphical assessment of the screeplot indicicate three factors. Factor 1 was interpreted as *Peaceful Acceptance* factor because the according items highly load on it. The items of the *Struggle with Illness* scale are distributed to factors 2 and 3, with PC 08 and PC 10 loading remarkably high on factor 2, PC 07, PC 09, PC 11, and PC 12 loading on the third factor, and PC 06 loading almost comparably on factor 2 and 3. The fact that most loadings on the pattern matrix are remarkably smaller than loadings on the structure matrix, showed that the factors are highly influencing each other (Watkins, 2018). The intercorrelation of the factors underlines this (please see Table S2-2).

**Figure S2-1:** *PAA - Screeplot*


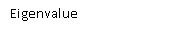


Factor

*Table S2-1: Structure Matrix (PAA, Promax rotation)*

|  | Factor | | |
| --- | --- | --- | --- |
|  | 1 | 2 | 3 |
| PC 01: Können Sie Ihre Krebsdiagnose akzeptieren? | .71 | -.54 | -.42 |
| PC 02: Empfinden Sie Ihrer Meinung nach inneren Frieden und Harmonie? | .79 | -.47 | -.54 |
| PC 03: Haben Sie Frieden mit Ihrer Erkrankung geschlossen? | .80 | -.61 | -.51 |
| PC 04: Fühlen Sie sich derzeit geliebt? | .29 | -.14 | -.24 |
| PC 05: Empfinden Sie innere Ruhe und Gelassenheit? | .69 | -.35 | -.46 |
| PC 06: Belasten Sie Veränderungen Ihres äußeren Erscheinungsbildes? | -.31 | .43 | .41 |
| PC 07: Hindert Sie die Sorge über Ihre Erkrankung, unbeschwert durch den Tag zu kommen? | -.55 | .44 | .57 |
| PC 08: Empfinden Sie es unfair, jetzt an Krebs erkrankt zu sein? | -.48 | .86 | .42 |
| PC 09: Haben Sie das Gefühl, dass Ihr Leben, so wie Sie es kennen, jetzt vorbei ist? | -.50 | .37 | .70 |
| PC 10: Sind Sie verärgert über Ihre Erkrankung? | -.51 | .77 | .47 |
| PC 11: Fühlen Sie sich von der Erkrankung in die Knie gezwungen? | -.53 | .44 | .76 |
| PC 12:. Schämen Sie sich für Ihren derzeitigen Gesundheitszustand bzw. ist Ihnen dieser peinlich? | -.34 | .37 | .49 |

*Table S2-2: Correlation matrix of factors (PAA, Promax rotation)*

|  | Factor | | |
| --- | --- | --- | --- |
| Factor | 1 | 2 | 3 |
| 1 | 1 | -.62 | -.64 |
| 2 | -.62 | 1 | .53 |
| 3 | -.64 | .53 | 1 |

*Table S2-3: Pattern matrix and explained variance (PAA, Promax rotation)*

|  | Factor | | |
| --- | --- | --- | --- |
|  | 1 | 2 | 3 |
| PC 01: Können Sie Ihre Krebsdiagnose akzeptieren? | .65 | -.19 | .10 |
| PC 02: Empfinden Sie Ihrer Meinung nach inneren Frieden und Harmonie? | .77 | .05 | -.07 |
| PC 03: Haben Sie Frieden mit Ihrer Erkrankung geschlossen? | .70 | -.21 | .05 |
| PC 04: Fühlen Sie sich derzeit geliebt? | .30 | .13 | -.11 |
| PC 05: Empfinden Sie innere Ruhe und Gelassenheit? | .72 | .13 | -.07 |
| PC 06: Belasten Sie Veränderungen Ihres äußeren Erscheinungsbildes? | .08 | .33 | .29 |
| PC 07: Hindert Sie die Sorge über Ihre Erkrankung, unbeschwert durch den Tag zu kommen? | -.28 | .08 | .34 |
| PC 08: Empfinden Sie es unfair, jetzt an Krebs erkrankt zu sein? | .08 | .91 | -.01 |
| PC 09: Haben Sie das Gefühl, dass Ihr Leben, so wie Sie es kennen, jetzt vorbei ist? | -.11 | -.05 | .66 |
| PC 10: Sind Sie verärgert über Ihre Erkrankung? | -.02 | .73 | .07 |
| PC 11: Fühlen Sie sich von der Erkrankung in die Knie gezwungen? | -.06 | .03 | .70 |
| PC 12: Schämen Sie sich für Ihren derzeitigen Gesundheitszustand bzw. ist Ihnen dieser peinlich? | .03 | .16 | .43 |
|  | % of total variance explained | | |
|  | 40.91 | 9.97 | 8.63 |
