# Supplementary material for: Peace, equanimity and acceptance in the cancer experience: validation of the German version (PEACE-G) and associations with mental health, health-related quality of life and psychological constructs
Source: BMC Psychol. 2024 Sep 27;12:507. doi: 10.1186/s40359-024-02018-8 (PMC11438294; doi:10.1186/s40359-024-02018-8)
Supplement: Supplementary file 3 — Supplementary Material 3 [file 40359_2024_2018_MOESM3_ESM.docx]

Supplement 3:

Sensitivity Analysis

We conducted several sensitivity analyses to investigate possible alternative model structures to improve the model fit. Therefore, an exploratory factor analysis was conducted. Following the guidelines of Watkins ^1^, a principal axis analysis (PAA) with least-square estimation method was used and the solution was rotated via Promax.

Following the PAA, a one-, bi-factor- and three-factor-model was tested with SEM. In search of possible alternatives to improve the model fit, the final model was calculated once with the subset of patients with metastatic cancer and once with the subset of patients with non-metastatic cancer, and both model fits were compared.

Results of sensitivity analysis

One-factor-model

Since factor 1 explains more than 40% of the variance and substantially more than factors 2 and 3, SEM was tested with one overall *Acceptance* factor explaining all the items. Results showed a worse fit than the two-factor solution; *χ^2^* (54) = 221.69, *p* < .001; CFI = 0.808; RMSEA = 0.121.

Three-factor-model

Based on the results of the PAA we tested a three-factor model. The model comprised a *Peaceful Acceptance* factor (PC 01 – PC 05), a second factor explaining PC 08 and PC 10, and a third factor with the other *Struggle with Illness* items (PC 06, PC 07, PC 09, PC 11, PC 12). The three-factor-model yielded better fit indices than the two-factor solution; *χ^2^* (51) = 116.08, *p* < .001; CFI = 0.925; RMSEA = 0.078. However, the slight improvement of indices comes at the price of reduced parsimony and reduced simplicity. The three-factor solution is depicted in Figure S3-1.

*Figure S3-1: Illustration of the three-factor-model*


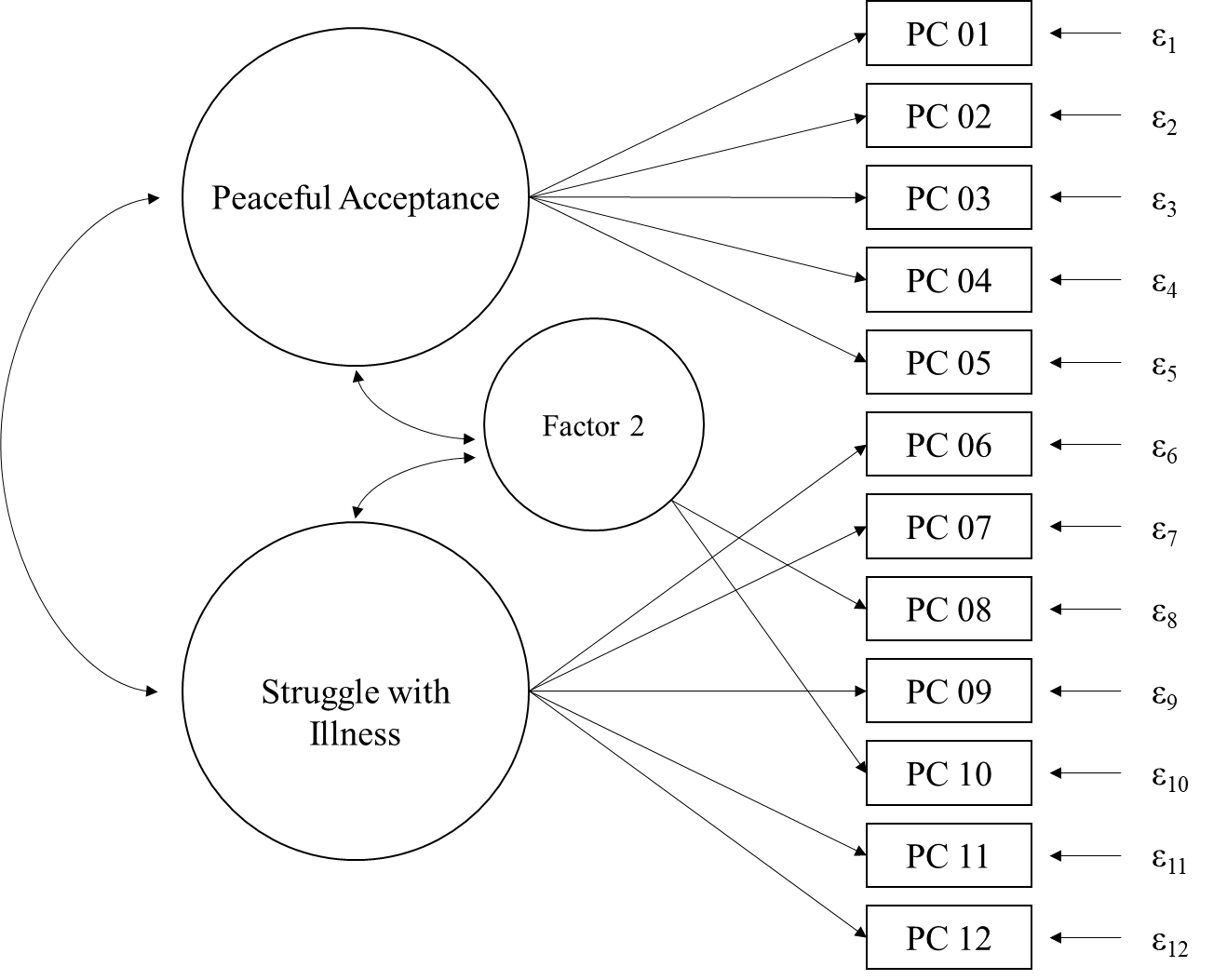


*Note.* The description of the additional factor as *Factor 2* refers to the results of the PAA, where this factor was the second factor extracted. In the [Discussion](#_Discussion), this factor is interpreted as an Injustice-*Anger* factor.

Bi-factor-model

As a combination of the one- and two-factor-model, a bi-factor-model was tested, which is illustrated in Figure S3-2. The model fit was slightly better regarding the χ^2^-test and CFI, while RMSEA was the same as the final two-factor-model; χ^2^ (42) = 100.29, *p* < .001; CFI = 0.933; RMSEA = 0.081. Just like the three-factor-model, this solution is less parsimonious and only improved some of the fit indices slightly, which is why it was not evaluated to fit the data better.

*Figure S3-2: Illustration of the Bi-factor-model*


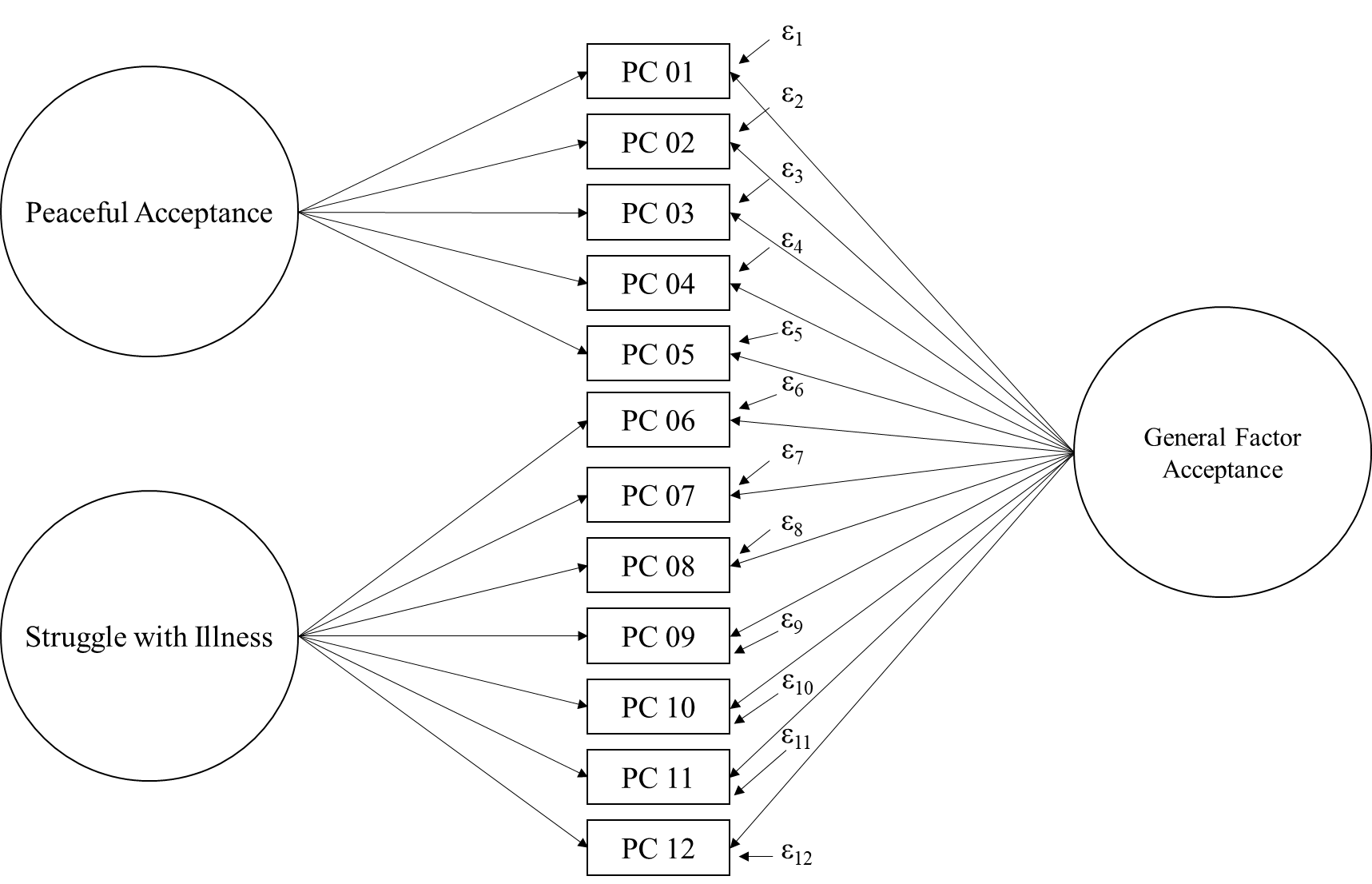


Since the two factors *Peaceful Acceptance* and *Struggle with Illness* highly correlate with each other, it was tested whether the phenomenon of vanishing factors biased the results of the bi-factor-model. Therefore, another bi-factor model (S-1) was calculated (see Figure S3-3). Results yielded a worse model fit (χ^2^ (47) = 153.38, *p* < .001; CFI = 0.878; RMSEA = 0.103), which is leading to the assumption that the phenomenon of vanishing factor didn’t bias the bi-factor solution.

Figure S3-3: Bi-factor (S-1) model

**
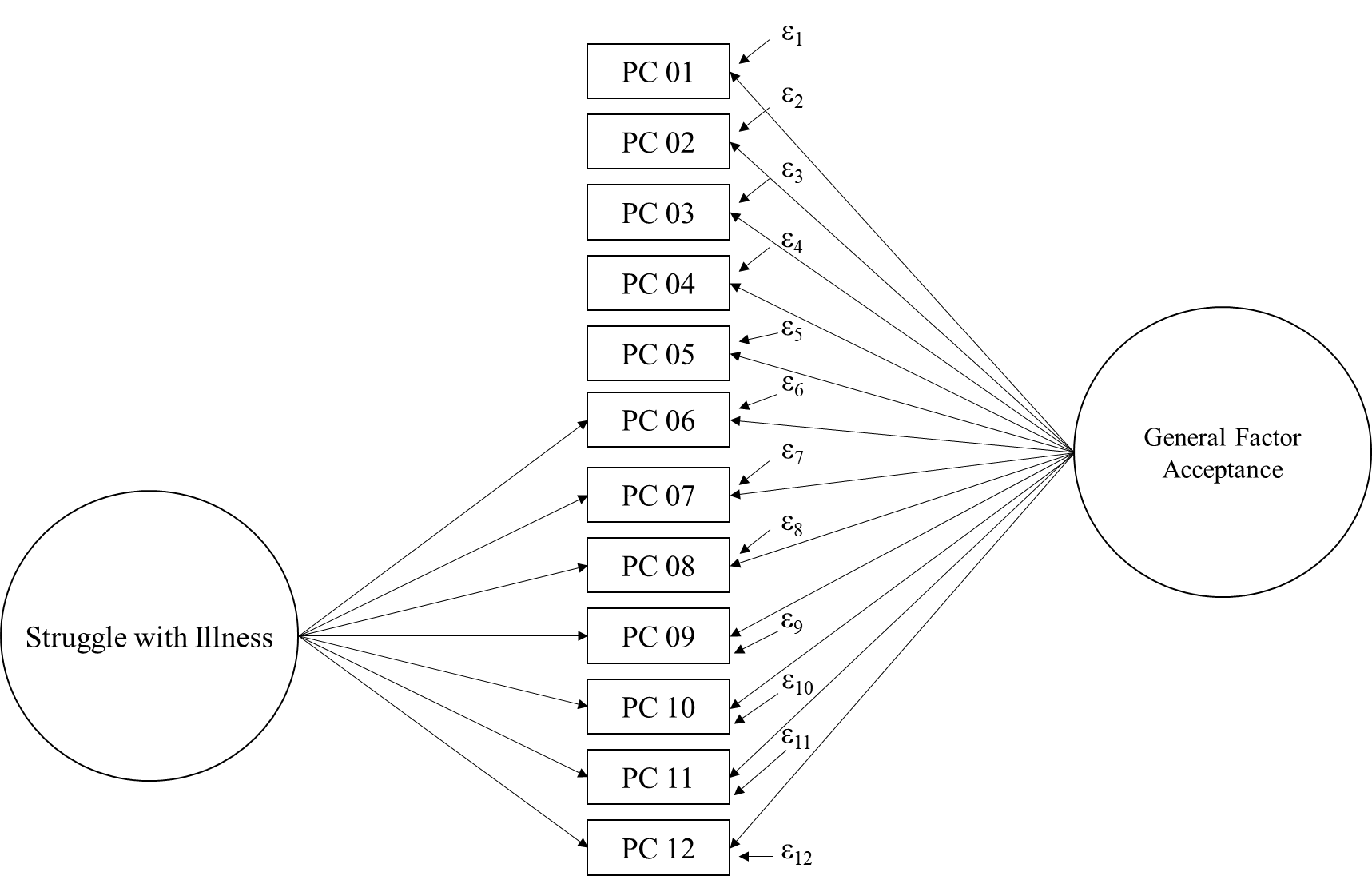
**

Metastatic vs. non-metastatic Cancer

The model fit within the subsample of patients with metastatic cancer and the model fit within the subsample of patients with non-metastatic cancer are shown in Table S3-1, just like an overview of all calculated models within sensitivity analysis. Results indicate that the model fit was substantially better in the subsample of patients with non-metastatic cancer. Furthermore, the improvement of model fit through integrating the covariance between residuals of PC 08 and PC 10 was remarkably higher within the subsample of patients with non-metastatic cancer (see also Table S3-1 ). To assess how item properties differ within the sample of metastatic and non-metastatic cancer, the item properties of each subsample were compared (Table S3-2). PC 08 and PC 10 correlated weaker with the total scale within the subsample of non-metastatic cancer; *r*(PC 08 | scale) = .52; *r*(PC 10 | scale) = .59; than in the subsample of metastatic cancer; *r*(PC 08 | scale) = .62; *r*(PC 10 | scale) = .63.

**Table S3-1**

*Overview of SEMs conducted in the course of sensitivity analyses*

|  | Index | | |
| --- | --- | --- | --- |
| Model | χ^2^ (df) | CFI | RMSEA |
| Original model^a^ | 168.04 (53) | 0.868 | 0.101 |
| Final model | 123.70 (52) | 0.918 | 0.081 |
| One-factor-model | 221.69 (54) | 0.808 | 0.121 |
| Three-factor-model | 116.08 (51) | 0.925 | 0.078 |
| Bi-factor-model | 100.29 (42) | 0.933 | 0.081 |
| Bi-factor (S-1) model | 153.38 (47) | 0.878 | 0.103 |
| Subsample metastatic cancer (final model; *n* = 126) | 119.32 (52) | 0.886 | 0.102 |
| Subsample non-metastatic cancer (final model; *n* = 87) | 68.26 (52) | 0.945 | 0.060 |
| Subsample metastatic cancer (model with independent residuals; *n* = 126) | 139.32 (53) | 0.854 | 0.114 |
| Subsample non-metastatic cancer (model with independent residuals; *n* = 87) | 96.00 (53) | 0.854 | 0.097 |

*Note.* ^a^Original model refers to the model with two correlated factors *Peaceful Acceptance* and *Struggle with Illness* with independent residuals which was tested first. The final model was then modified by integrating a covariance between residuals of PC 08 and PC 10. Each χ^2^-test was significant at a level of *p* < .001.

*Table S3-2: Item properties of the subsample with non-metastatic and metastatic cancer*

|  | Subsample non-metastatic cancer (*n* = 87) | | Subsample metastatic cancer  (*n* = 126)^a^ | |
| --- | --- | --- | --- | --- |
|  | *r* (Item \| Subscale) | Mean (*SD*) | *r* (Item \| Subscale) | Mean (*SD*) |
| 1. Können Sie Ihre Krebsdiagnose akzeptieren? | .58 | 3.35 (0.66) | .62 | 3.11 (0.88) |
| 2. Empfinden Sie Ihrer Meinung nach inneren Frieden und Harmonie? | .65 | 2.95 (0.86) | .67 | 3.00 (0.82) |
| 3. Haben Sie Frieden mit Ihrer Erkrankung geschlossen? | .61 | 3.08 (0.80) | .74 | 2.92 (0.99) |
| 4. Fühlen Sie sich derzeit geliebt? | .23 | 3.70 (0.59) | .26 | 3.79 (0.53) |
| 5. Empfinden Sie innere Ruhe und Gelassenheit? | .40 | 3.01 (0.76) | .63 | 3.05 (0.80) |
| 6. Belasten Sie Veränderungen Ihres äußeren Erscheinungsbildes? | .46 | 2.63 (1.01) | .42 | 2.27 (0.97) |
| 7. Hindert Sie die Sorge über Ihre Erkrankung, unbeschwert durch den Tag zu kommen? | .52 | 2.32 (0.83) | .61 | 2.44 (0.91) |
| 8. Empfinden Sie es unfair, jetzt an Krebs erkrankt zu sein? | .52 | 2.52 (1.08) | .62 | 2.53 (1.12) |
| 9. Haben Sie das Gefühl, dass Ihr Leben, so wie Sie es kennen, jetzt vorbei ist? | .54 | 2.26 (1.04) | .57 | 2.56 (1.05) |
| 10. Sind Sie verärgert über Ihre Erkrankung? | .59 | 2.66 (1.12) | .63 | 2.74 (1.07) |
| 11. Fühlen Sie sich von der Erkrankung in die Knie gezwungen? | .52 | 2.26 (1.02) | .66 | 2.48 (1.04) |
| 12. Schämen Sie sich für Ihren derzeitigen Gesundheitszustand bzw. ist Ihnen dieser peinlich? | .39 | 1.54 (0.89) | .50 | 1.44 (0.69) |
| *Note.* ^a^Two participants with missings were excluded, resulting in *n* = 124 for this calculation. | | | | |

Reference:

1. Watkins MW. Exploratory factor analysis: A guide to best practice. J Black Psychol. 2018;44(3):219-46.
