# Supplementary material for: Peace, equanimity and acceptance in the cancer experience: validation of the German version (PEACE-G) and associations with mental health, health-related quality of life and psychological constructs
Source: BMC Psychol. 2024 Sep 27;12:507. doi: 10.1186/s40359-024-02018-8 (PMC11438294; doi:10.1186/s40359-024-02018-8)
Supplement: Supplementary file 4 — Supplementary Material 4 [file 40359_2024_2018_MOESM4_ESM.docx]

**Supplement 4: Results of Cross-validation**

*Fit indices of the original and modified model within different compositions of subsamples*

|  | Model with independent residuals | | |  | Model with covariance between residuals of PC 08 and PC 10 | | |
| --- | --- | --- | --- | --- | --- | --- | --- |
|  | χ^2^ (df) | CFI | RMSEA |  | χ^2^ (df) | CFI | RMSEA |
| A: Subsample 1 | 118.05 (53) | 0.859 | 0.106 |  | 90.85 (52) | 0.916 | 0.082 |
| A: Subsample 2 | 110.06 (53) | 0.861 | 0.103 |  | 92.30 (52) | 0.902 | 0.088 |
| B: Subsample 1 | 107.43 (53) | 0.894 | 0.098 |  | 91.78 (52) | 0.922 | 0.085 |
| B: Subsample 2 | 107.56 (53) | 0.840 | 0.099 |  | 89.27 (52) | 0.890 | 0.083 |
| C: Subsample 1 | 117.89 (53) | 0.823 | 0.107 |  | 85.76 (52) | 0.908 | 0.078 |
| C: Subsample 2 | 103.83 (53) | 0.896 | 0.096 |  | 89.49 (52) | 0.923 | 0.083 |
| D: Subsample 1 | 116.88 (53) | 0.841 | 0.106 |  | 103.55 (52) | 0.872 | 0.096 |
| D: Subsample 2 | 133.40 (53) | 0.834 | 0.121 |  | 97.77 (52) | 0.906 | 0.092 |

*Note.* Method A = Sample was split chronologically into the first and second subsample. Method B = Each second pair of two participants was assigned to another subsample. Method C = Each second pair of three participants was assigned to another subsample. Method D = Each second pair of four participants was assigned to another subsample. Each χ^2^-test was significant at a level of *p* < .001.
