# Supplementary material for: Peace, equanimity and acceptance in the cancer experience: validation of the German version (PEACE-G) and associations with mental health, health-related quality of life and psychological constructs
Source: BMC Psychol. 2024 Sep 27;12:507. doi: 10.1186/s40359-024-02018-8 (PMC11438294; doi:10.1186/s40359-024-02018-8)
Supplement: Supplementary file 5 — Supplementary Material 5 [file 40359_2024_2018_MOESM5_ESM.docx]

Supplement 5: Reliability parameters of the PEACE-G

|  | Cronbach‘s α | *r* (Item \| Subscale) | *M* (*SD*) | Cronbach’s α without item |
| --- | --- | --- | --- | --- |
| *Peaceful Acceptance* Scale^a^ | .79 |  |  |  |
| 1. Können Sie Ihre Krebsdiagnose akzeptieren? |  | .63 | 3.21 (0.80) | .74 |
| 2. Empfinden Sie Ihrer Meinung nach inneren Frieden und Harmonie? |  | .70 | 2.98 (0.84) | .71 |
| 3. Haben Sie Frieden mit Ihrer Erkrankung geschlossen? |  | .70 | 2.98 (0.92) | .71 |
| 4. Fühlen Sie sich derzeit geliebt? |  | .26 | 3.75 (0.56) | .83 |
| 5. Empfinden Sie innere Ruhe und Gelassenheit? |  | .58 | 3.04 (0.78) | .75 |
| *Struggle with Illness* Scale | .79 |  |  |  |
| 6. Belasten Sie Veränderungen Ihres äußeren Erscheinungsbildes? |  | .43 | 2.42 (1.00) | .78 |
| 7. Hindert Sie die Sorge über Ihre Erkrankung, unbeschwert durch den Tag zu kommen? |  | .52 | 2.39 (0.88) | .77 |
| 8. Empfinden Sie es unfair, jetzt an Krebs erkrankt zu sein? |  | .57 | 2.53 (1.10) | .76 |
| 9. Haben Sie das Gefühl, dass Ihr Leben, so wie Sie es kennen, jetzt vorbei ist? |  | .52 | 2.43 (1.05) | .77 |
| 10. Sind Sie verärgert über Ihre Erkrankung? |  | .59 | 2.70 (1.09) | .75 |
| 11. Fühlen Sie sich von der Erkrankung in die Knie gezwungen? |  | .58 | 2.39 (1.03) | .76 |
| 12. Schämen Sie sich für Ihren derzeitigen Gesundheitszustand bzw. ist Ihnen dieser peinlich? |  | .45 | 1.48 (0.78) | .78 |

*Note.* *N* = 213. ^a^ *N = 211. M* = Mean, *SD* = Standard deviation.
